# First-generation bypass surgery for a giant fusiform aneurysm of the middle cerebral artery: an illustrative case and surgical video

**DOI:** 10.1093/jscr/rjae083

**Published:** 2024-02-22

**Authors:** Jhon E Bocanegra-Becerra, José Luis Acha Sánchez

**Affiliations:** School of Medicine, Universidad Peruana Cayetano Heredia, Lima, Peru; Department of Neurosurgery, Hospital Nacional Dos de Mayo, Lima, Peru; Universidad Nacional Mayor de San Marcos , Lima, Peru

**Keywords:** giant fusiform aneurysm, cerebral bypass, cerebral revascularization, microsurgery, STA-MCA

## Abstract

Giant fusiform aneurysms of the middle cerebral artery (MCA) are complex and rare vascular lesions with a poor natural history and challenging treatment decision-making. We report the case of a 46-year-old male with a history of chronic hypertension and a transient ischemic attack who presented with left-sided hemiparesis. A cerebral angiotomography revealed an unruptured giant fusiform aneurysm in the M2 segment of the right MCA. After carefully evaluating the procedure’s risks and benefits with the patient, he underwent a low-flow bypass surgery. An anastomosis between the superficial temporal artery and the M3 segment was performed with proximal clipping of the M2 segment. The postoperative course was uneventful, with preserved bypass patency. At follow-up, the patient was neurologically intact. This report illustrates the nuances and operative techniques for treating a giant fusiform aneurysm of the M2 segment that accounted for a preserved bypass patency and optimal patient neurological recovery.

## Introduction

Fusiform aneurysms are elongated and spindle-like-shaped lesions involving a large segment of an affected artery [1]. When a marked concentric dilation of the vessel is greater than 2.5 cm, it constitutes a giant-sized fusiform aneurysm [2]. These vascular lesions are rare and represent ~5%–17.6% of giant aneurysms [3]. Although commonly reported in the vertebrobasilar system, autopsy studies suggest a similar prevalence in the anterior circulation, including the middle cerebral artery (MCA) territory [2, 4, 5].

The natural history of giant fusiform aneurysms is often poor and can be characterized by thrombosis, growth, and catastrophic rupture [5]. Furthermore, aneurysms associated with intracranial atherosclerosis present with a worse prognosis [6]. According to Seo *et al.*, patients with fusiform aneurysms and segmental ectasia located in the post-bifurcation of the MCA are more likely to present with hemorrhagic symptoms [7]. Hence, in this morphological type, the authors suggested that blood pressure control is paramount if observation without surgical intervention is considered [7].

Treatment decision-making of giant MCA fusiform aneurysms is challenging, given a complex, diseased architecture that could compromise vital arterial branches and perforators. Evolving strategies, including endovascular and microsurgical approaches, have been proposed; the latter, exemplified by aneurysm clipping and cerebral revascularization techniques, demands thoughtful contemplation of the aneurysm morphology, compromised MCA segment, and the patient’s history and comorbidities [8, 9].

In this case, we illustrate the technical nuances that accounted for cerebral revascularization of the MCA through a first-generation bypass with preserved patency and optimal patient neurological recovery, thus supporting the technique’s viability and long-standing potential for select giant MCA fusiform aneurysms.

## Case presentation

**Figure 1:**
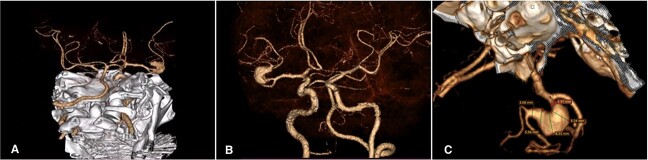
Cerebral Angiotomography. (A) Posterior view depicts an unruptured giant fusiform aneurysm in the M2 segment of the right MCA; (B) Anterior view reveals a close aneurysm proximity to the M1 segment of 3 mm; (C) Complex architecture of the giant fusiform aneurysm is shown with adjacent M2 and M3 branches. MCA: middle cerebral artery

A 46-year-old male with a past medical history of arterial hypertension presented with a 4-month history of recurrent and sporadic headaches, transient ischemic stroke, and a recent 3-day course of left hemiparesis. Neurological examination showed mild 4/5 left-sided hemiparesis and a Glasgow coma scale (GCS) score of 15. A subsequent cerebral angiotomography revealed an unruptured 25 × 10 mm fusiform aneurysm in the superior M2 segment of the MCA (Fig. 1). After discussing potential risks, benefits, and natural history with the patient, we decided to perform a superficial temporal artery (STA) to M3 bypass with proximal occlusion of the giant fusiform aneurysm.

### Operative note

**Figure 2:**
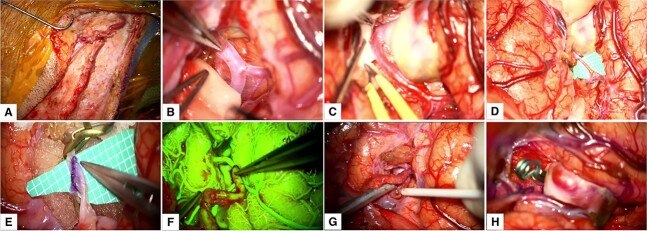
Intraoperative Course. (A) Harvesting of the STA; (B) Distal dissection of the Sylvian fissure to expose the M2 segment; (C) Proximal dissection of the Sylvian fissure exposed the M1 segment, M2 bifurcation and the giant fusiform aneurysm; (D) and (E) M3 segment arteriotomy and end-to-side anastomosis between the STA and MCA; (F) and (G) Intraoperative fluorescein imaging and Doppler utilization to corroborate bypass patency; (H) Permanent clipping and exclusion of the giant fusiform aneurysm of the superior M2 segment. MCA: middle cerebral artery, STA: superficial temporal artery.

The patient was placed supine, with table elevation at 20° and head rotation at 30°. Graft harvesting was achieved by fine dissection of the right STA from the zygomatic arch level until its frontal and parietal branches, averaging 8 cm in length (Video S1 and Fig. 2). The graft was then clipped proximally and washed with a heparinized solution in preparation for anastomosis. Scalp dissection was continued with diligent preservation of facial nerve branches, followed by a pterional craniotomy (not shown in the video). Distal Sylvian fissure dissection exposed the M2 and M3 segments and the distal portion of the giant fusiform aneurysm. Then, we dissected the Sylvian fissure proximally to reveal the carotid cistern and identified the internal carotid artery at the A1-M1 bifurcation and the M1 segment course. Once the proximal M2 segment was found, we visualized the origin of the aneurysm (Video S1 and Fig. 2).

Upon complete exposure of the Sylvian fissure, we applied temporal clips at the distal M2 segment. Careful arteriotomy of the M3 segment was done. Next, an end-to-side anastomosis was performed between the STA and M3 segment. Proximal and distal temporary clips were removed to evaluate the patency of the STA-MCA bypass, which was later corroborated by the use of fluorescein angiography and intraoperative Doppler. Finally, the giant fusiform aneurysm was excluded from circulation by placing a permanent clip at the proximal M2 segment (Video S1 and Fig. 2).

### Postoperative course

**Figure 3:**
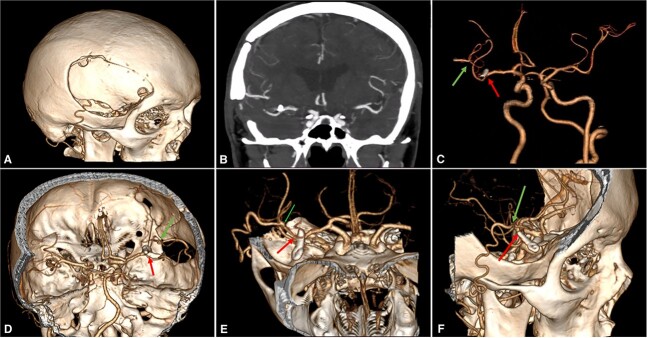
Postoperative Imaging. (A) 3-Dimensional image reconstruction shows the craniotomy with the entrance of the STA into the skull; (B) Coronal view of a maximum intensity projected multislice spiral CT shows the permanent clip placed with the exclusion of the fusiform aneurysm; (C)–(F) Cranial views depict patency of the STA-MCA bypass (green arrow) and clipping of the M2 proximal segment (red arrow). CT: computed tomography, MCA: middle cerebral artery, STA: superficial temporal artery.

Postoperative brain imaging showed adequate bypass patency with aneurysm exclusion from the circulation (Video S1 and Fig. 3). The patient was discharged 13 days later with a GCS score of 15 and a modified Rankin Score (mRS) of 1. At the 6-month follow-up, the patient was neurologically intact (mRS: 0).

## Discussion

Giant MCA fusiform aneurysms are complex vascular lesions that could be further complicated by the presence of intraluminal thrombi, atherosclerotic plaques, mural calcifications, and the involvement of branches arising from the aneurysm sac [10].

Treatment decision demands careful preoperative planning and study of the affected vessel, the patient’s clinical history, and comorbidities [10, 11]. Surgical management, including clipping and revascularization techniques, offers durable and effective treatment. For instance, the STA-MCA bypass has been a long-standing resource with high patency rates (86.7–97%) and optimal clinical outcomes [11–17]. Although emerging endovascular procedures (i.e. flow diversion) and combined microsurgical and endovascular approaches have been proposed, some authors argue microsurgical intervention is amenable for MCA aneurysms and superior to endovascular treatment [18–21]. On the other hand, no Class 1 evidence compares endovascular treatment with surgical treatment for giant/fusiform aneurysms that could guide better decision-making [22]. A notable algorithm for cerebral revascularization of MCA aneurysms has been proposed by Tayebi Meybodi *et al.*, which suggests treatment according to the location of the aneurysm in the MCA (i.e. pre-bifurcation, bifurcation, and post-bifurcation areas), lenticulostriate anatomy, and rupture status. For aneurysms located in the post-bifurcation and close to the insular recess—like in our case—the authors suggest revascularization with an STA-MCA bypass and proximal occlusion instead of trapping/excision to preserve the efferent arteries buried in the insular recess [21].

In the presented case, the preoperative study of the intricated vessel anatomy was deemed favorable for microsurgical intervention. By using a low-flow extracranial-to-intracranial bypass and proximal occlusion, this technique was able to exclude the aneurysm from circulation and preserve the efferent arteries from the territory contiguous to the fusiform aneurysm, which were difficult to visualize in the operative field. Consequently, these technical nuances ensured the optimal neurological recovery of the patient.

## Conclusion

Giant MCA fusiform aneurysms are rare and complex lesions demanding a comprehensive appraisal of the diseased vessel, branches, and patient’s clinical history. In the evolving landscape of treatment strategies, first-generation bypass surgery has been a long-standing resource to offer revascularization of the affected brain territory with high patency rates and good clinical results. This report illustrated the nuances and operative techniques for treating a giant fusiform aneurysm of the M2 segment that accounted for a preserved bypass patency and optimal patient neurological recovery at follow-up.

## Supplementary Material

Video_1_rjae083
